# Reversible changes in pancreatic islet structure and function produced by elevated blood glucose

**DOI:** 10.1038/ncomms5639

**Published:** 2014-08-22

**Authors:** Melissa F. Brereton, Michaela Iberl, Kenju Shimomura, Quan Zhang, Alice E. Adriaenssens, Peter Proks, Ioannis I. Spiliotis, William Dace, Katia K. Mattis, Reshma Ramracheya, Fiona M. Gribble, Frank Reimann, Anne Clark, Patrik Rorsman, Frances M. Ashcroft

**Affiliations:** 1 Henry Wellcome Centre for Gene Function, Department of Physiology, Anatomy and Genetics and OXION, University of Oxford, Parks Road, Oxford OX1 3PT, UK; 2 Oxford Centre for Diabetes, Endocrinology and Metabolism, University of Oxford, Churchill Hospital, Oxford OX3 7LE, UK; 3 Cambridge Institute for Medical Research, Addenbrooke’s Hospital, Hills Road, Cambridge CB2 0XY, UK; 4 These authors contributed equally to this work

## Abstract

Diabetes is characterized by hyperglycaemia due to impaired insulin secretion and aberrant glucagon secretion resulting from changes in pancreatic islet cell function and/or mass. The extent to which hyperglycaemia *per se* underlies these alterations remains poorly understood. Here we show that β-cell-specific expression of a human activating K_ATP_ channel mutation in adult mice leads to rapid diabetes and marked alterations in islet morphology, ultrastructure and gene expression. Chronic hyperglycaemia is associated with a dramatic reduction in insulin-positive cells and an increase in glucagon-positive cells in islets, without alterations in cell turnover. Furthermore, some β-cells begin expressing glucagon, whilst retaining many β-cell characteristics. Hyperglycaemia, rather than K_ATP_ channel activation, underlies these changes, as they are prevented by insulin therapy and fully reversed by sulphonylureas. Our data suggest that many changes in islet structure and function associated with diabetes are attributable to hyperglycaemia alone and are reversed when blood glucose is normalized.

## Abstract (web summary)

In patients with diabetes, insulin release from pancreatic β-cells is reduced due to altered islet structure and function. Here, Brereton *et al*. show that elevated blood glucose underlies these changes and is sufficient to reversibly alter β-cell identity in a mouse model of β-cell dysfunction.

## Graphical abstract

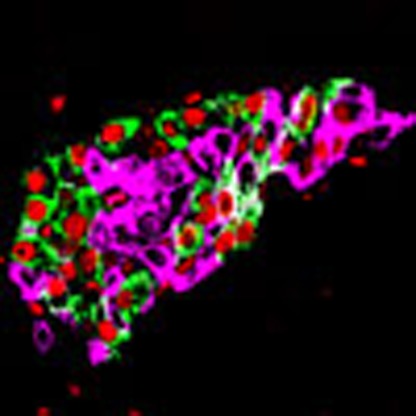

Diabetes mellitus currently affects over 330 million people worldwide. It is a heterogeneous disorder with multiple aetiologies, but all result in a persistently elevated blood glucose concentration as a consequence of insufficient insulin release by pancreatic β-cells^1^. In type-1 diabetes, the insulin-producing β-cells are destroyed and patients require lifelong treatment with exogenous insulin. In type-2 diabetes mellitus (T2DM), the β-cells largely remain, but they fail to release sufficient insulin to maintain normoglycaemia. Both reduced β-cell mass and impaired β-cell function have been proposed to underlie the defective insulin secretion, but their relative contribution is debated^2^,^3^,^4^. There are also a number of rare monogenic forms of diabetes that present either at birth (neonatal diabetes) or in young adult life (maturity onset diabetes of the young)^1^. The primary problem in almost all of these genetic disorders is insufficient insulin secretion. In addition, glucagon secretion from pancreatic α-cells is usually disturbed.

The extent to which hyperglycaemia *per se* underlies, or exacerbates, alterations in β-cell and α-cell function and/or mass in diabetes remains poorly understood. Numerous studies have examined the effect of hyperglycaemia on isolated islets, β-cells or β-cell lines^5^. These have concluded that culture in high glucose (>20 mM) for several days leads to a reduction in insulin content, impaired insulin secretion and multiple changes in gene expression. However, such studies have the limitation that changes in gene expression may occur as a consequence of *in vitro* culture. The effects of hyperglycaemia have also been explored using various mouse models of diabetes^6^. Most of these, however, suffer from the disadvantage that the insulin secretory defect cannot be reversed, either because the diabetes is genetic or is artificially induced by β-cell ablation. Furthermore, the diabetogenic gene(s) may be unknown, is usually expressed in all tissues and may be associated with insulin resistance and/or obesity. This makes dissecting the effect of hyperglycaemia *per se* on pancreatic islet cells *in vivo* difficult. In this respect, a means of selectively and reversibly switching off insulin secretion would be advantageous. One way to do so is by manipulating ATP-sensitive K^+^ (K_ATP_) channel activity.

The prominent role of the K_ATP_ channel in regulating glucose-stimulated insulin secretion is well established^7^,^8^. These channels are regulated by metabolically generated ATP and thereby link changes in blood glucose concentration to insulin release. When K_ATP_ channels are open, as at low plasma glucose levels, Ca^2+^ influx and insulin secretion are prevented, switching off insulin release. A rise in blood glucose elevates intracellular ATP, closing K_ATP_ channels and leading to membrane depolarization, β-cell electrical activity, Ca^2+^ influx and insulin release. The crucial role of K_ATP_ channel function in controlling blood glucose is illustrated by the fact that mutations in K_ATP_ channel genes can cause aberrant insulin secretion. For example, gain-of-function mutations in either the Kir6.2 or SUR1 subunits of the K_ATP_ channel are a common cause of neonatal diabetes, a rare genetic form of the disease that presents within the first 6 months of life^9^,^10^,^11^. All these mutations impair the ability of metabolically generated ATP to close the channel, and thereby prevent insulin secretion. Mice that express activating K_ATP_ channel mutations selectively in their pancreatic β-cells recapitulate many of the characteristics of neonatal diabetes^12^,^13^,^14^: in particular, they display hyperglycaemia and hypoinsulinaemia in the absence of obesity and insulin resistance.

Here we use a transgenic mouse that expresses a human neonatal diabetes mutation (Kir6.2-V59M) specifically in β-cells^12^ to investigate the effects of chronic hyperglycaemia on islet cell structure and function. In this mouse model, insulin secretion is rapidly switched off following K_ATP_ channel activation but can be restored by treatment with sulphonylurea (SU) drugs, which are specific K_ATP_ channel blockers. This allowed us to assess the effects of reversible hyperglycaemia on pancreatic islet cells. Our results suggest that many of the changes in islet structure and function associated with diabetes are attributable to hyperglycaemia, and can be reversed by normalization of blood glucose. These data have implications for understanding the aetiology and treatment of diabetes.

## Results

### Expression of Kir6.2-V59M in β-cells rapidly induces diabetes

**Figure 1:**
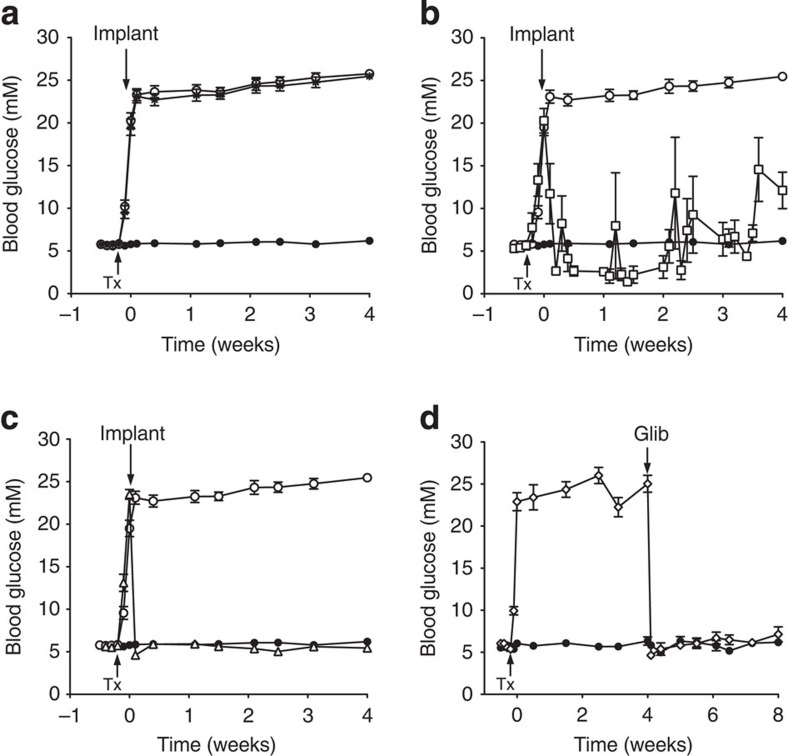
Gene induction results in rapid diabetes that is normalized by insulin and SU therapy. (**a**) Blood glucose levels for 12-week-old βV59M (open circle, black star) and control (black circle, *n*=41) mice. Mice were injected with tamoxifen (Tx) as indicated by the arrow to induce Kir6.2-V59M expression. Some Tx-injected mice were subsequently implanted with a subcutaneous slow-release placebo pellet at time zero (black star, *n*=35), whereas others were not (open circle, *n*=31). (**b**,**c**) Blood glucose levels measured in βV59M mice injected with Tx (arrow) and subsequently implanted (arrow) with an insulin pellet (**b**; open square, *n*=6) or glibenclamide pellet (**c**; open triangle, *n*=19). Control mice (black circle) and Tx-induced untreated βV59M mice (open circle) are the same data as in Fig. 1a. (**d**) βV59M mice injected with Tx (arrow) and subsequently implanted (arrow) with a glibenclamide pellet (Glib) after 4 weeks of diabetes (open diamond, *n*=6). Control littermates (black circle, *n*=6) were sham injected with Tx. Data are mean values±s.e.m.

We generated an inducible mouse model selectively expressing a gain-of-function K_ATP_ channel mutation (Kir6.2-V59M) in pancreatic β-cells (βV59M mice). Expression was induced at 12 weeks of age by tamoxifen injection. This resulted in a rapid rise in the blood glucose concentration of free-fed mice that exceeded 20 mM within 2 days and was sustained throughout the next 4 weeks (Fig. 1a). Free-fed (Supplementary Fig. 1a) and fasted (Supplementary Fig. 1b) plasma insulin levels were significantly reduced (~60%) by Kir6.2-V59M gene expression. Plasma glucagon levels did not change (Supplementary Fig. 1c,d).

We examined the effects of insulin or glibenclamide therapy using slow-release pellets implanted subcutaneously once blood glucose had risen above 20 mM. Free-fed blood glucose levels were not significantly different in βV59M mice implanted with a placebo pellet (containing no drug) from those that did not receive a pellet (Fig. 1a).

Implantation of an insulin pellet (releasing 0.2–0.3U per day) caused a rapid fall in blood glucose from 20.3±1.4 mM (*n*=6) on the day of implantation to 8.2±3.2 mM 2 days later (Fig. 1b). However, insulin therapy failed to maintain normoglycaemia consistently throughout the 4-week period, and sporadic episodes of hypoglycaemia (<2 mM) and hyperglycaemia (>20 mM) were common.

Glycaemic control was greatly improved in mice treated with glibenclamide pellets (Fig. 1c). However, the dose needed to control the blood glucose level varied with the duration of hyperglycaemia. When glibenclamide was administered immediately following a rise in blood glucose to >20 mM, a dose of 17 mg kg^−1^ per day was sufficient to produce normoglycaemia (Fig. 1c), whereas a dose of 34–95 mg kg^−1^ per day was necessary to normalize glycaemia in mice that had been diabetic for 4 weeks (Fig. 1d).

Glibenclamide therapy caused a rapid fall in free-fed blood glucose: 2 days after drug implantation, glucose concentrations were 4.6±0.2 mM (*n*=19; Fig. 1c) in mice previously exposed to 24 h of hyperglycaemia and 4.6±0.4 mM (*n*=6; Fig. 1d) in those exposed to 4 weeks of hyperglycaemia. These values are close to those measured in the same animals before gene induction (5.6±0.1 mM; *n*=25) or in control mice (5.9±0.2 mM; *n*=41). Blood glucose levels were stable throughout the 4 weeks of glibenclamide treatment.

### Effect of long-term hyperglycaemia on islet morphology

**Figure 2:**
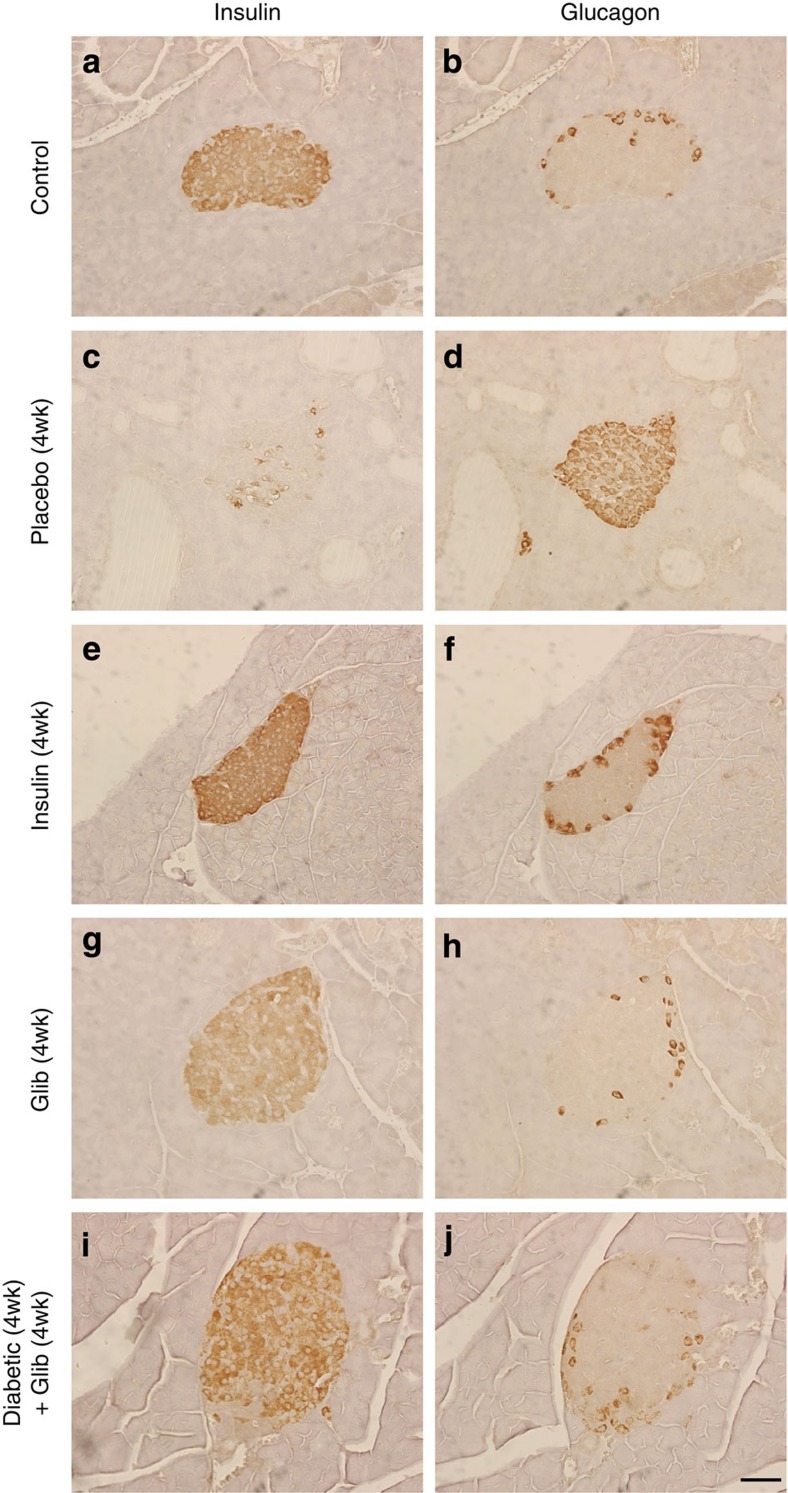
Chronic hyperglycaemia alters insulin and glucagon immunostaining in pancreatic islets. Representative serial sections of mouse pancreas immunostained for insulin (left) or glucagon (right) using DAB (brown). Control mouse pancreas (**a**,**b**). βV59M mouse pancreas 4 weeks after implantation with a placebo (**c**,**d**), insulin (**e**,**f**), or glibenclamide (**g**,**h**) pellet. (**i**,**j**) Islets from βV59M mice exposed to 4 weeks of hyperglycaemia and then 4 weeks of glibenclamide therapy (*n*=4). Results are representative of four (**a**–**d**,**i**–**j**) or three (**g**,**h**) mice. Scale bar, 50 μm (applies to all panels).

**Figure 3:**
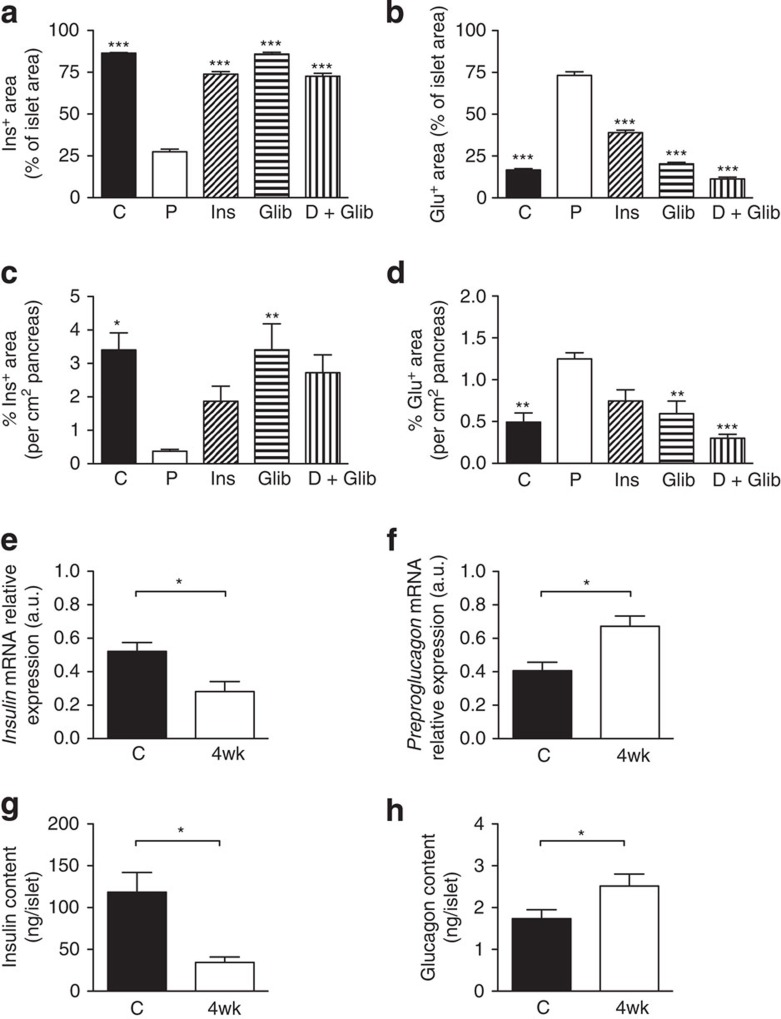
Effects of chronic hyperglycaemia on insulin and glucagon levels. Mean islet cross-sectional area immunostaining for insulin (**a**,**c**) or glucagon (**b**,**d**), expressed either as a percentage of the total islet cross-sectional area (**a**,**b**) or per cm^2^ of pancreas (**c**,**d**). Once plasma glucose exceeded 20 mM, βV59M mice were treated for 4 weeks with placebo (P), insulin (Ins) or glibenclamide (Glib); or, following 4 weeks of no therapy, with 4 weeks of glibenclamide (D+Glib). Data are mean±s.e.m. of three to six mice per genotype (five sections per mouse, 100 μm apart). (*P<0.05; **P<0.01, ***P<0.001 compared with placebo (P); one-way analysis of variance followed by *post-hoc* Bonferroni test). Insulin (**e**) and preproglucagon (**f**) mRNA levels determined by qPCR in islets isolated from control (C, black bars) and 4-week-diabetic (4wk, white bars) βV59M mice (*n*=6–7 per genotype). Insulin (**g**) and glucagon (**h**) protein content of islets determined by radioimmunoassay from control (C) and 4-week-diabetic (4wk) βV59M mice (*P<0.05; Mann–Whitney test; *n*=6–7 per genotype). Data are mean values±s.e.m.

Hyperglycaemia for 4 weeks led to marked changes in islet morphology. There was a dramatic decrease in insulin-positive (ins^+^) cells and a concomitant increase in glucagon-positive (glu^+^) cells, which were no longer confined to the mantle but populated the core of the islet (compare Fig. 2c,d with Fig. 2a,b). Quantitative analysis revealed a marked reduction (~70%) in the area of the islet staining for insulin and an equivalent increase in the area staining for glucagon (Fig. 3a,b). A reduction in the percentage area of the pancreas staining for insulin (a surrogate for β-cell mass) and an increase in the area staining for glucagon was also observed (Fig. 3c,d). Chronic hyperglycaemia did not produce a statistically significant change in the percentage area of the pancreas occupied by islets (Supplementary Fig. 2a) or in islet density (islet number per cm^2^ pancreas) (Supplementary Fig. 2b,c).

Insulin therapy largely prevented the reduction in insulin staining and increase in glucagon staining when data were expressed relative to islet area (Fig. 2e,f and Fig. 3a,b) or per cm^2^ pancreas (Fig. 3c,d). Thus, the decline in ins^+^ cells (and corresponding increase in glu^+^ cells) is principally the result of hyperglycaemia *per se* and not K_ATP_ channel hyperactivity.

Glibenclamide therapy also prevented the diabetes-induced changes in insulin (Fig. 2g) and glucagon (Fig. 2h) staining, and in the area of individual islets, or whole pancreas, composed of ins^+^ and glu^+^ cells (Fig. 3a–d). Drug treatment was slightly more effective than insulin, perhaps because it produced more stable control of blood glucose (compare Fig. 1b and c).

Remarkably, 4 weeks of glibenclamide therapy almost fully reversed the histological changes produced by 4 weeks of hyperglycaemia. Insulin staining was observed throughout the islet and glucagon staining was once more confined to the mantle (Fig. 2i,j). The islet area (Fig. 3a,b) and pancreas area (Fig. 3c,d) occupied by ins^+^ or glu^+^ cells were restored to levels similar to those found in control mice.

Consistent with the decrease in the percentage of the islet area showing insulin immunoreactivity, and the concomitant increase in glucagon immunoreactivity, insulin messenger RNA and insulin content were reduced (Fig. 3e,g), and preproglucagon mRNA and glucagon content were enhanced (Fig. 3f,h), in islets isolated from mice that had been hyperglycaemic for 4 weeks, when compared with control animals.

### Effects of hyperglycaemia on islet cell ultrastructure

**Figure 4:**
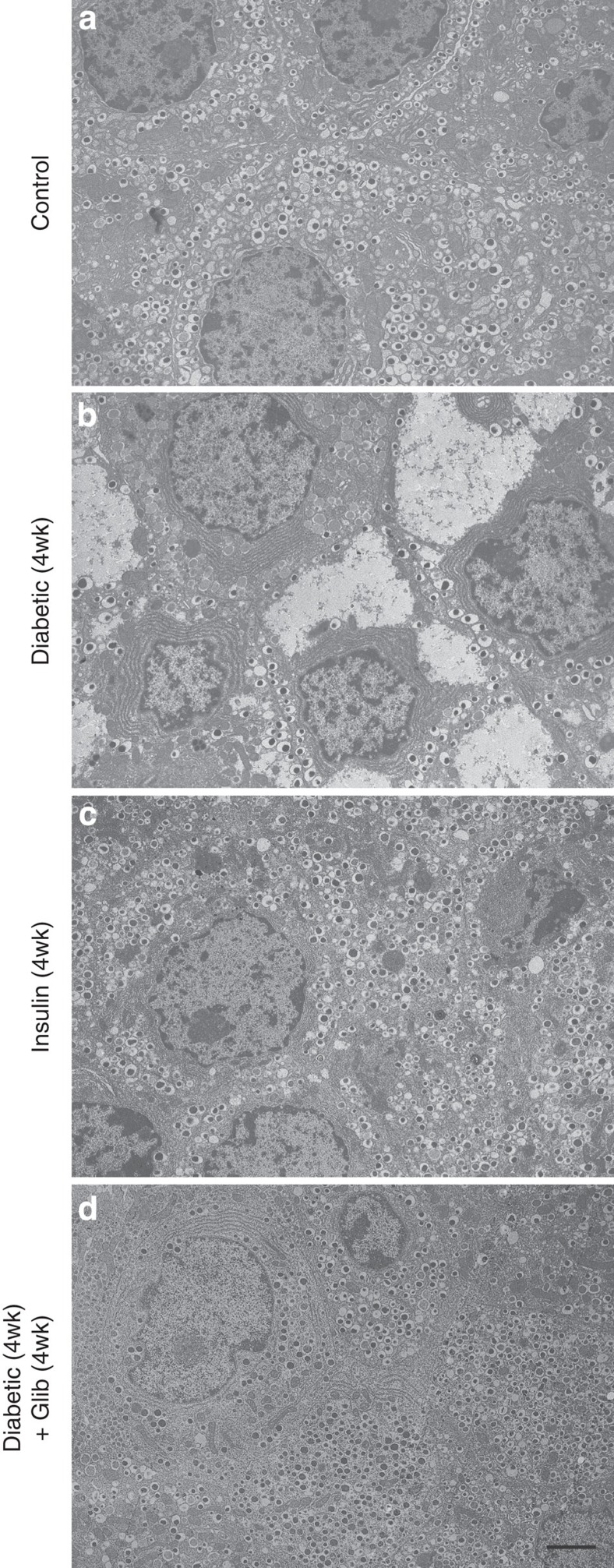
Chronic hyperglycaemia reversibly alters β-cell ultrastructure. Representative electron micrographs of pancreatic sections from control mice (**a**), βV59M mice exposed to hyperglycaemia for 4 weeks (**b**), βV59M mice treated with insulin for 4 weeks (**c**) and βV59M mice that were hyperglycaemic for 4 weeks and then treated with glibenclamide for 4 weeks (**d**). Scale bar, 2 μm (refers to all panels). Images are representative of: **a**, 9 mice/9 islets/150 β-cells; **b**, 3 mice/9 islets/134 β-cells; **c**, 2 mice/3 islets/100 β-cells; **d**, 3 mice/3 islets/105 β-cells.

Exposure to chronic diabetes resulted in striking changes in β-cell ultrastructure. Compared with control littermates (Fig. 4a), the number of insulin granules was considerably reduced in mice exposed to diabetes for 4 weeks (Fig. 4b). Surprisingly, in many β-cells, large areas of cytoplasm were filled with a homogeneous unstructured substance that displaced the intracellular organelles. The shape of the nucleus was occasionally distorted, but there was no indication of any apoptotic changes. No morphological signs of cellular stress were detected: the mitochondria and endoplasmic reticulum were not swollen and appeared normal. Furthermore, transcript levels of the endoplasmic reticulum (ER) stress markers *Chop* and spliced *Xbp*1 were unaltered in islets isolated from 4-week diabetic mice compared with controls (Supplementary Fig. 3a). The ultrastructural changes were specific to β-cells, and not observed in adjacent α-cells or δ-cells (Supplementary Fig. 3b).

No effect on β-cell ultrastructure was observed following Kir6.2-V59M gene induction and only 24 h of hyperglycaemia and β-cells remained well granulated (Supplementary Fig. 3c). This supports the view that K_ATP_ channel activation acutely inhibits insulin release by preventing β-cell electrical activity^12^,^14^ rather by affecting insulin content.

Immediate insulin therapy prevented the ultrastuctural changes associated with diabetes (Fig. 4c). Even more remarkably, the ultrastructural changes associated with 4 weeks of diabetes were completely reversed after 4 weeks of glibenclamide therapy (Fig. 4d). In glibenclamide-treated islets, the majority of β-cells were densely packed with insulin granules, which had a size and morphology similar to those observed in control animals. Taken together, the data indicate that β-cell ultrastructural changes are due to hyperglycaemia and/or hypoinsulinaemia and—importantly—are reversible on restoration of euglycaemia.

### Effect of chronic hyperglycaemia on islet cell identity

We next investigated the mechanism(s) underlying the alterations in islet cell composition in chronically diabetic βV59M mice. We first explored whether they were caused by changes in cell turnover. Because of marked β-cell degranulation in βV59M mice, we used electron microscopy to identify β-cells unequivocally and nuclear morphology (condensed chromatin) as a measure of apoptosis. We found 3.7% of β-cells (that is, cells containing typical insulin granules) from 4-week diabetic βV59M mice had apoptotic nuclei (Supplementary Fig. 3d); similar numbers were found in control islets (4.0%). Cell proliferation, determined by Ki67 positivity, was also unchanged in glu^+^ cells (Supplementary Fig. 4a,b). Thus, the changes we observe are unlikely to be due to β-cell death or α-cell proliferation.

**Figure 5:**
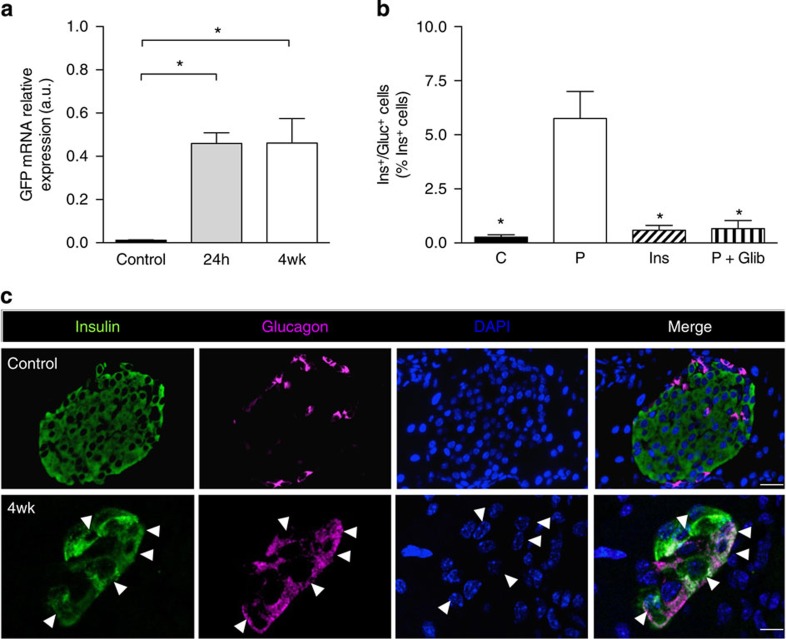
Insulin/glucagon double-positive cells are reversibly increased by hyperglycaemia. (**a**). GFP mRNA levels determined by qPCR in islets isolated from control mice and βV59M mice 24-h (24 h) and 4 weeks (4wk) after diabetes onset. Data are mean values±s.e.m., *n*=4 per genotype. (*P<0.05; one-way analysis of variance (ANOVA) followed by *post-hoc* Bonferroni test). (**b**) Dual ins^+^/glu^+^ cells expressed as a percentage of the total number of ins^+^ cells. Control islets (C). Islets from βV59M mice implanted with placebo (P) or insulin (Ins) for 4 weeks, or treated for 4 weeks with glibenclamide after 4 weeks of hyperglycaemia (P+Glib). Data are mean values±s.e.m., *n*=2,600–7,700 ins^+^ cells; *n*=92–127 islets; *n*=3–4 mice per genotype. (*P<0.05 compared with placebo (P); one-way ANOVA followed by *post-hoc* Bonferroni test). (**c**) Representative example of immunofluorescence staining for insulin (green), glucagon (pink), DAPI (4′,6-diamidino-2-phenylindole; blue) and merged data (white) in control islets and 4-week βV59M diabetic islets. White arrowheads indicate cells positive for both insulin and glucagon. Scale bars, 50 μm control; 10 μm 4-week βV59M diabetic islets.

We also examined transcript levels of green fluorescent protein (GFP), which is located downstream of Kir6.2-V59M in the transgene cassette and thus serves as a marker of gene induction^12^. A >30-fold increase in islet GFP mRNA was observed 24 h after establishment of a free-fed blood glucose of >20 mM by gene induction (Fig. 5a). Islet GFP mRNA levels were unchanged after 4 weeks of chronic hyperglycaemia (Fig. 5a), despite the reduction in islet insulin immunostaining (Fig. 2), insulin mRNA (~50%, Fig. 3e) and insulin content (~70%, Fig. 3g). This provides further support for the idea that the reduction in ins^+^ cells we observe is not due to β-cell death.

Chronic hyperglycaemia was associated with a ~20-fold increase in cells positive for both insulin and glucagon (ins^+^/glu^+^ cells) in islets from 4-week-diabetic βV59M mice (Fig. 5b,c). However, in mice treated with insulin for 4 weeks immediately after establishment of diabetes, or treated with glibenclamide following 4 weeks of hyperglycaemia, the number of ins^+^/glu^+^ cells was not significantly different from control animals (Fig. 5b). This argues the increase in ins^+^/glu^+^ cells is caused by hyperglycaemia/hypoinsulinaemia and is reversed when blood glucose is normalized.

**Figure 6:**
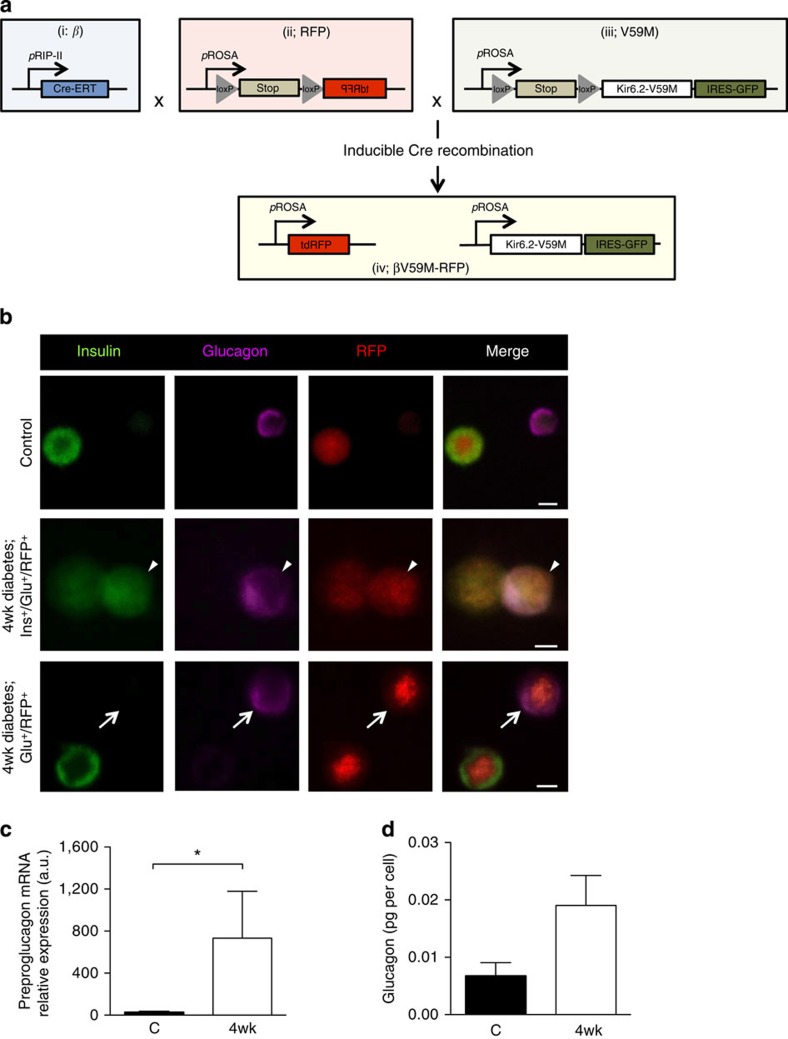
Chronic hyperglycaemia induces glucagon expression in β-cells. (**a**) Schematic illustrating how *Rip-CreER*^*+/+*^ *(i)*, *Rosa*^*RFP/*−^ *(ii)* and *Rosa*^*V59M/*−^ *(iii)* were used to generate *Rosa*^*RFP/V59M*^ mice (βV59M-RFP mice). β-Cells were selectively and irreversibly labelled following tamoxifen injection, by crossing an inducible rat insulin promoter Cre line (i; β) with a floxed tdRFP reporter line in which tdRFP expression was driven by the endogenous ROSA promoter (ii; RFP). These β-RFP control mice were then crossed with an inducible Kir6.2-V59M line (iii; V59M) to create βV59M-RFP mice (iv). (**b**) Representative examples of immunofluorescence staining for insulin (green), glucagon (pink) and RFP (red) in control (top panel, β-RFP) and 4-week-diabetic βV59M-RFP (middle and bottom panels) isolated islet cells. White arrows, RFP^+^/glu^+^ cells. White arrowhead, RFP^+^/glu^+^/ins^+^ cell. Scale bar, 10 μm. (**c**,**d**) Islet cells from β-RFP and 4-week-diabetic βV59M-RFP mice were FAC-sorted into RFP^+^ and RFP^−^ populations, and analysed for preproglucagon mRNA by qPCR (**c**) and glucagon protein (**d**); *n*=4 mice per genotype. Data are mean values±s.e.m. *P<0.05; Mann–Whitney test.

We used lineage tracing to test whether β-cells start to express glucagon when exposed to chronic hyperglycaemia. Although GFP was located downstream of Kir6.2-V59M in the transgene cassette, expression was too weak to be visualized by immunofluorescence. Thus, a Rosa26^RFP^ reporter was crossed into RIPII-CreER mice with (βV59M-RFP mice) or without (β-RFP control mice) the floxed Kir6.2-V59M transgene, to label all β-cells and their progeny with red fluorescent protein (RFP) after tamoxifen injection (Fig. 6a and Supplementary Fig. 5a,b).

In β-RFP mice, 67% of ins^+^ cells labelled with RFP (this indicates the recombination frequency). In βV59M-RFP mice exposed to chronic hyperglycaemia for 4 weeks, 7% of RFP^+^ cells (that is, cells of β-cell lineage) contained both insulin and glucagon, and 8% expressed glucagon alone (Fig. 6b and Supplementary Fig. 5c). Approximately 60% of RFP^+^ cells expressed insulin and 24% did not detectably express either insulin or glucagon. Fluorescence-assisted cell (FAC) sorting by RFP fluorescence (Supplementary Fig. 5a,b) revealed RFP^+^ cells from diabetic βV59M-RFP mice had more preproglucagon mRNA (Fig. 6c) and glucagon protein (Fig. 6d), and less insulin content (Supplementary Fig. 5d), than β-RFP littermate controls. No significant change in glucagon content was observed in RFP^−^ cells (Supplementary Fig. 5e).

**Figure 7:**
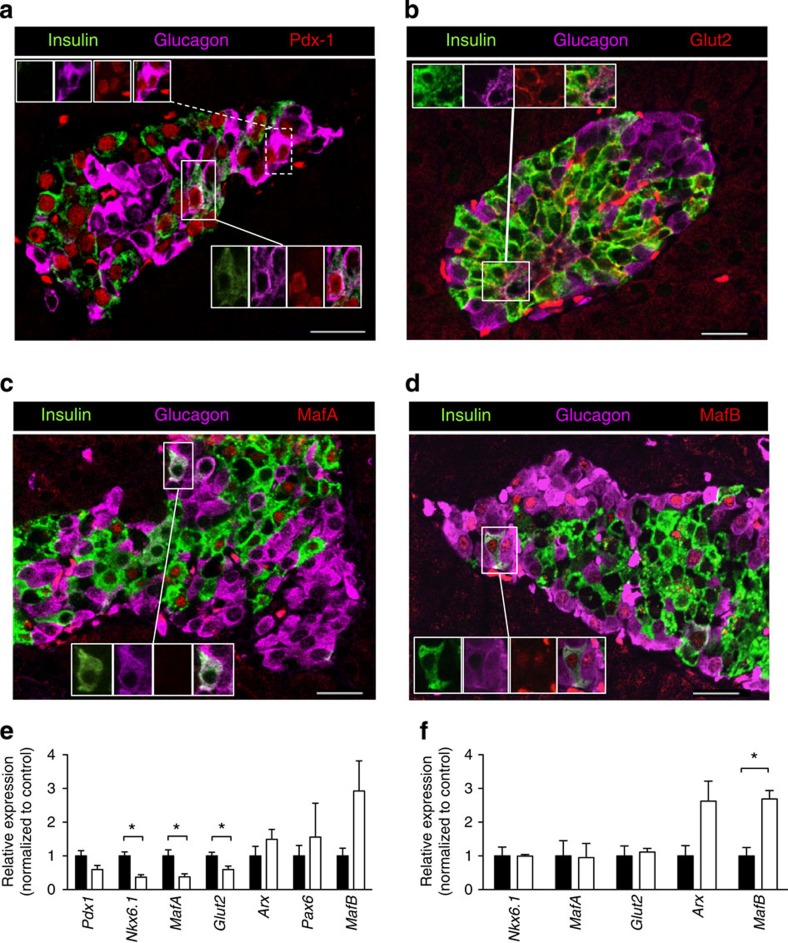
Effects of hyperglycaemia on islet cell transcription factors and transporters. (**a**–**d**) Representative examples of immunofluorescence staining for (red) Pdx-1 (**a**), Glut2 (**b**), MafA (**c**) or MafB (**d**) in a 4-week-diabetic βV59M islet. Insulin (green), glucagon (pink). (**a**) Insets show (upper) glu^+^ and (lower) ins^+^/glu^+^ cells that express Pdx1. (**b**) Inset shows an ins^+^/glu^+^ cell that expresses Glut2. (**c**) Inset shows an ins^+^/glu^+^ cell that does not express MafA. (**d**) Inset shows an ins^+^/glu^+^ cell that expresses MafB. Scale bars, 50 μm. (**e**) *Pdx-1, Nkx6.1, MafA*, *Glut2, Arx, Pax6* and *MafB* mRNA levels assessed by qPCR in islets isolated from control mice (black bars) and 4-week-diabetic βV59M mice (white bars). Data are mean values±s.e.m., *n*=6–7 mice per genotype. (*P<0.05; Mann–Whitney test). (**f**) *Nkx6.1, MafA, Glut2, Arx* and *MafB* mRNA levels in FAC-sorted RFP^+^ cells isolated from control mice (black bars) and 4-week-diabetic βV59M mice (white bars). Data are mean values±s.e.m., *n*=4 mice per genotype. (*P<0.05; Mann–Whitney test).

Expression of several β-cell (Pdx-1, MafA, Nkx6.1 and Glut2) and α-cell (Arx, Pax6 and MafB) markers was examined to determine whether the molecular identity of islet cells was altered by exposure to chronic hyperglycaemia. Immunofluorescence microscopy of intact islets from 4-week-diabetic βV59M mice identified expression of Pdx-1 (Fig. 7a), Glut2 (Fig. 7b) and MafA (Fig. 7c) in ins^+^ cells. Ins^+^/glu^+^ cells retained expression of Pdx-1 and Glut2, but also expressed MafB (Fig. 7d). All glu^+^ cells expressed MafB but a few also contained Pdx1. Nkx6.1 protein was undetectable by immunofluorescence in 4-week diabetic islets (compare Supplementary Fig. 6a with 6b). Quantitative PCR (qPCR) analysis revealed reduced expression of the β-cell markers *Pdx-1* (*P*=0.05; Mann-Whitney test), *Nkx6.1, MafA* and *Glut2* in intact islets from 4-week-diabetic βV59M mice (Fig. 7e). Although the α-cell marker *MafB* appeared elevated, *Arx* and *Pax6* mRNA levels were unchanged (Fig. 7e).

In contrast to what was observed in islets, mRNA levels of *Nkx6.1*, *MafA* and *Glut2* were unaltered in FAC-sorted RFP^+^ β-cells (Fig. 7f). The difference between the effect of diabetes on mRNA expression in islets and FAC-purified RFP^+^ β-cells is probably due to loss of a specific β-cell subpopulation during FAC sorting: ~30% fewer RFP^+^ β-cells were FAC sorted from 4-week diabetic mice than from β-RFP mice. It is possible that the absent cells represent β-cells with large areas of unstructured cytoplasm (identified in electron microscopy), which may be more fragile and thus do not survive FAC sorting.

Expression of the α-cell transcription factors *MafA* and *Arx* was increased in RFP^+^ FAC-sorted β-cells (Fig. 7f), which explains the elevated glucagon content. Taken together, the data confirm that cells with a β-cell lineage express glucagon in response to chronic hyperglycaemia by increasing expression of α-cell transcription factors. In addition, the islet progenitor cell marker *Ngn3* was elevated in both islets (Supplementary Fig. 6b) and FAC-sorted RFP^+^ β-cells from 4-week-diabetic βV59M mice (Supplementary Fig. 6c).

**Figure 8:**
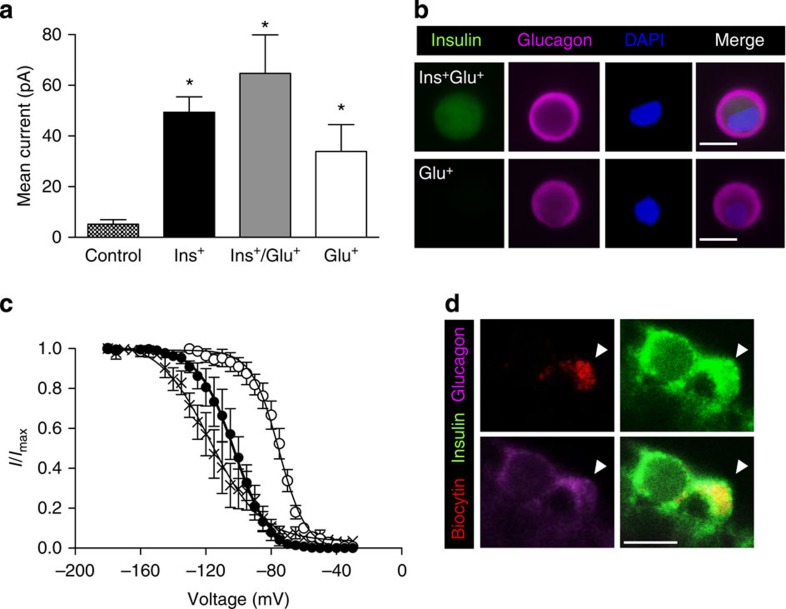
Glucagon-expressing β-cells retain β-cell electrophysiological characteristics. (**a**) Mean±s.e.m. cell-attached K_ATP_ currents from control β-cells (hatched bars; *n*=27) and from 4-week-diabetic βV59M islet cells that express insulin (black bars; *n*=48), insulin and glucagon (grey bars; *n*=5), or glucagon alone (white bars; *n*=6). (*P<0.05; one-way analysis of variance followed by *post-hoc* Bonferroni test). (**b**) Representative examples of cells exhibiting large K_ATP_ currents obtained in cell-attached patches that expressed both insulin and glucagon (top panels) or glucagon alone (bottom panels). Scale bar, 10 μm. (**c**) Voltage-dependent inactivation of whole-cell Na^+^ currents in β-cells (closed circles; *n*=5) and α-cells (open circles; *n*=6) from control mice and in ins^+^/glu^+^ cells from 4-week-diabetic βV59M mice (crosses; *n*=6). The pulse protocol consisted of 1 ms depolarizations to 0 mV preceded by 200 ms conditioning pulses to membrane potentials between −180 and −5 mV. Data are mean values±s.e.m. The superimposed curves represent Boltzmann fits to the data. (**d**) Representative example of a patched cell (indicated by the white arrowhead) showing Na^+^ current inactivation characteristic of a β-cell and identified by infusion of biocytin (red, upper left panel) that expressed both insulin (green, upper right) and glucagon (pink, lower left). Merged images, lower right panel. Scale bar, 10 μm.

We also assessed the electrophysiological fingerprint of islet cells from control and βV59M mice following 4 weeks of diabetes. All cells were identified by immunolabelling for insulin and glucagon after patch clamping. In βV59M mice, the Cre-lox approach restricts expression of the mutant Kir6.2 subunit to cells expressing insulin at the time of induction. As the Kir6.2-V59M mutation markedly decreases K_ATP_ channel inhibition by ATP, and thereby increases current amplitude^12^,^15^, large amplitude K_ATP_ currents also serve as a lineage marker for β-cells. Cell-attached K_ATP_ currents from control β-cells were small (5±2 pA; *n*=27), due to inhibition by intracellular ATP. In contrast, ins^+^ cells from βV59M mice had ~10-fold larger currents (49±6 pA; *n*=48). Large on-cell K_ATP_ currents were also recorded from ins^+^/glu^+^ cells, and even from a number of cells that expressed glucagon alone (Fig. 8a,b).

Marked differences in the voltage-dependence of Na^+^-current inactivation were observed between control mouse β- and α-cells in intact freshly isolated islets, with half-maximal inactivation (*V*_0.5_) occurring at −103±1 mV (*n*=5) and −75±1 mV (*n*=6), respectively (Fig. 8c). In ins^+^/glu^+^ cells (Fig. 8c,d), *V*_0.5_ was −117±2 mV (*n*=6; also measured in acutely isolated intact islets): this is similar to control β-cells and provides further evidence that ins^+^/glu^+^ cells derive from β-cells and retain many β-cell properties.

## Discussion

Our data demonstrate that expression of a human activating K_ATP_ channel mutation in adult mouse β-cells leads to rapid diabetes and marked alterations in islet morphology and ultrastructure. Following 4 weeks of diabetes, islet insulin mRNA and protein were dramatically reduced and glucagon mRNA and protein increased. Importantly, the reduced insulin staining seen with light microscopy did not equate with loss of β-cells, as evident from electron microscopy. Some β-cells now expressed glucagon and several α-cell transcription factors. However, they retained β-cell characteristic proteins such as Glut2 and β-cell voltage-gated Na^+^ channels. We show that hyperglycaemia, rather than K_ATP_ channel activation *per se*, accounts for these changes, as they can be prevented by insulin therapy. Strikingly, the effects of chronic hyperglycaemia were also reversed when blood glucose was normalized with the SU glibenclamide.

K_ATP_ channel activation led to a rapid and sustained rise in blood glucose on gene induction. Treatment of βV59M mice with either insulin or glibenclamide normalized blood glucose within 24 h of implementing therapy. Glibenclamide produced far more stable control of plasma glucose than insulin (as it does in patients with neonatal diabetes^16^). This may be because SUs exert their effect indirectly by stimulating insulin release from pancreatic β-cells^17^. K_ATP_ channel closure by SUs enables additional mechanisms that enhance meal-induced insulin secretion, such as the stimulatory effects of incretins (triggered by the presence of food in the gut^18^) and the non-K_ATP_-dependent actions of glucose^19^. This should help reduce plasma glucose fluctuations by directly linking insulin release to plasma glucose concentrations and food ingestion. In contrast, insulin therapy results in large spikes in blood glucose (as also seen in βV59M mice), because an insulin dose sufficient to fully control meal-stimulated spikes in plasma glucose cannot be used, as it would produce hypoglycaemia at fasting plasma glucose levels.

Surprisingly, glibenclamide therapy rapidly normalized plasma glucose levels, even after 4 weeks of diabetes. This agrees with clinical data: many patients with neonatal diabetes have been able to transfer to SU therapy, albeit with a very high dose, even after years of insulin treatment^18^,^20^. Similar to patients with many years of poor glycaemic control, βV59M mice required higher drug doses following 4 weeks, rather than 2 days, of hyperglycaemia. This may explain previous reports that glibenclamide was unable to reverse established diabetes in mice with a different activating K_ATP_ channel mutation, which used a lower drug dose^14^,^21^.

Dramatic changes in insulin content, islet morphology and β-cell ultrastructure were evident after 4 weeks of diabetes. There was a marked reduction in islet cells staining for insulin, consistent with the fall in islet insulin mRNA and protein levels. Nevertheless, our data indicate that the loss of insulin immunolabelling did not equate to loss of β-cell mass. This is clear from the fact that many β-cells, albeit with very few insulin granules, are observed at the ultrastructural level. The nuclear morphology of these β-cells also confirms they are not undergoing apoptosis. Furthermore, islet GFP mRNA levels do not decrease with diabetes duration, as would be expected if β-cells die (GFP serves as a β-cell lineage marker in βV59M mice). We also found no change in apoptosis. Thus, our data argue that the marked decrease in insulin staining we observe by immunohistochemistry is not primarily due to β-cell loss but rather to a dramatic reduction in insulin gene expression and insulin granule density. The idea that reduced insulin content can give rise to the fallacious impression of β-cell loss has also been suggested for islets from patients with type T2D (ref. 22) and rodent models of diabetes^23^.

In our mouse model, chronic hyperglycaemia not only led to a remarkable reduction in insulin granule content but also to the appearance of large areas of unstructured cytoplasm within β-cells. We saw no morphological evidence of increased numbers of lysosomes or autophagic bodies, and these cells did not show any morphological signs of apoptotic death or ER stress. Furthermore, the structural changes were fully reversible on administration of glibenclamide; hence, they are not a sign of permanent β-cell damage. Such dramatic changes in β-cell ultrastructure have not been reported previously, either in mouse models of diabetes or in islets from patients with T2DM. Thus, it is possible they arise from a combination of hyperglycaemia/hypoinsulinaemia and K_ATP_ channel activation.

Chronic hyperglycaemia was also associated with a marked increase in preproglucagon mRNA and glucagon content, as well as glucagon immunostaining, in β-cells. However, the increase in glucagon immunostaining was significantly less than the reduction in insulin immunostaining, when normalized to pancreatic area. This is consistent with the presence of many sparsely granulated β-cells that do not contain sufficient insulin for detection by conventional immunostaining. The low levels of glucagon in β-cells (average ~0.02 pg/RFP^+^ cell) compared with pre-existing α-cells (~1.5 pg/RFP^−^ cell) (Fig. 6d and Supplementary Fig. 5d) may explain why there was no obvious change in plasma glucagon despite the increase in glu^+^ cells and islet glucagon content. Furthermore, glucagon expressed by β-cells is unlikely to be released due to the hyperpolarizing effect of the K_ATP_ channel mutation.

Lineage tracing and electrophysiological recordings confirmed that ins^+^/glu^+^ cells and a number of cells expressing glucagon alone were of β-cell origin, as they expressed RFP and displayed very large K_ATP_ currents (indicating they carried the Kir6.2-V59M mutation). These cells also retained several β-cell functional characteristics; they expressed the β-cell transcription factor Pdx-1, the β-cell glucose transporter Glut2 and the voltage-dependence of Na^+^ current inactivation was characteristic of β-cell, not α-cell, Na^+^ channels^24^,^25^. However, the α-cell transcription factors *MafB* and *Arx* were upregulated in RFP^+^ β-cells, which probably explains their increased glucagon expression^26^,^27^.

In previous studies, insulin and glucagon content, and/or islet cell transcription factors have commonly been used to define β-cell or α-cell identity. What constitutes a β-cell may be a matter of semantics; nevertheless, our results raise the question of whether transcription factor expression and insulin/glucagon content are sufficient to define β-cell identity. In our mouse model, β-cells (identified by lineage tracing) showed lower insulin and increased glucagon content following 4 weeks of diabetes. However, they maintained a partial β-cell identity; they expressed Pdx1 and Glut2, and had Na^+^ channels characteristic of β-cells (not α-cells).

Several studies have highlighted the remarkable plasticity of islet cells. β-Cells can transdifferentiate into α-cells^23^,^27^,^28^,^29^,^30^. Similarly, conversion of α-cells into β-cells following near-total β-cell ablation has been observed^31^ and alterations in the expression and activity of key β-cell transcription factors, including Nkx6.1 (refs 32, 33, 34, 35), FoxO1 (ref. 23) and Pdx-1 (ref. 35), are commonly observed following dedifferentiation or transdifferentiation of islet cells. Chronic hyperglycaemia in both diabetic rodent models and human type 2 diabetic islets can also lead to changes in transcription factor expression and the appearance of ins^+^/glu^+^ cells^23^,^31^,^33^,^34^,^35^,^36^. Our data consolidate these findings by showing that hyperglycaemia *per se* can drive alterations in islet cell identity.

A recent study of mice with an activating K_ATP_ channel mutation (ΔN30-K185E) also found that hyperglycaemia led to a dramatic loss of insulin content without marked changes in islet cell death or proliferation^37^. They attributed this to β-cell dedifferentiation due to upregulation of the islet progenitor marker Ngn3. Although we found a similar increase in *Ngn3* mRNA, we were unable to observe significant protein expression. Wang *et al*.^37^ also reported a marked increase in glucagon immunostaining but found few glu^+^ cells that were of β-cell lineage. One possible reason for these differences may be that β-cells from ΔN30-K185E mice might have dedifferentiated further than those of βV59M mice, perhaps because of the higher blood glucose level of ΔN30-K185E mice (>33 versus 25 mM). How the severity and duration of hyperglycaemia affects β-cell plasticity is currently unknown. Thus, we cannot exclude the possibility that the changes we observe reflect an early intermediary state in β-cell dedifferentiation or transdifferentiation to α-cells. However, at this stage, their glucose transporters and Na^+^ channels are more characteristic of β-cells than α-cells or islet progenitor cells.

Our results suggest many of the changes we observed in islet morphology and ultrastructure can be attributed to hyperglycaemia rather than K_ATP_ channel activation. Insulin therapy, which normalizes blood glucose without affecting the open K_ATP_ channel, prevented the changes in insulin- and glucagon-staining, β-cell ultrastructure and number of ins^+^/glu^+^ cells produced by 4 weeks of diabetes. Furthermore, normalization of blood glucose by glibenclamide following 4 weeks of diabetes reversed these changes, suggesting that β-cell functional mass can be immediately restored when blood glucose is controlled. This may explain why glucose-induced insulin secretion is reinstated in patients with T2DM given a low-calorie diet^38^. Although we cannot unequivocally distinguish between the effects of hyperglycaemia and hypoinsulinaemia, the fact that deletion of the insulin receptor in β-cells^39^ does not alter their ultrastructure, or the relative proportions of ins^+^ and glu^+^ cells, favours the former possibility.

In conclusion, we show that chronic hyperglycaemia results in the partial loss of β-cell identity in a mouse model of β-cell dysfunction. Strikingly, these alterations are prevented and reversed when blood glucose is normalized with anti-diabetic drugs. This highlights the remarkable plasticity of β-cells and their ability to reversibly alter their gene expression, structure and function in response to changes in circulating glucose levels. Our results also highlight the importance of good glucose control in patients with diabetes and indicate that this should help preserve β-cell structure and function, and may even reverse established changes.

## Methods

### Generation of βV59M mice

All experiments were conducted in accordance with the UK Animals Scientific Procedures Act (1986) and have been approved by the University of Oxford DPAG local ethical committee. Mice hemizygously expressing Kir6.2-V59M in insulin-secreting cells (βV59M mice) were generated using a Cre-lox approach essentially as described^12^ but using an inducible rat insulin promoter II (RIPII-Cre-ER mice^40^). Kir6.2-V59M expression was induced in 12-to 14-week-old male and female mice by a single subcutaneous injection of 0.4 ml of 20 mg ml^−1^ tamoxifen in corn oil (Sigma). All experiments were performed in 12- to 14-week-old mice with a mixed (C3H, C57BL/6, 129/sv) genetic background. Wild-type mice, RIPII-Cre-ER mice and mice expressing only the floxed Kir6.2-V59M gene (ROSA) were used as controls. *Rosa*^*RFP*+/−^ mice were produced as described^41^.

### Molecular biology

Total RNA was prepared from isolated islets, amplified using the Ovation Pico WTA System V2 (NuGen) kit and reversed transcribed. qPCR was performed as described^42^ and transcript levels of *Arx*, *Chop*, *Gfp*, *glucagon*, *Glut2*, *insulin*, *MafA*, *MafB*, *Ngn3*, *Nkx6.1*, *Pax6*, *Pdx1* and *Xbp1* spliced and three reference genes (*Actb*, *Hprt1* and *Hspa8*) quantified (for primer sequences see Supplementary Table 1). Similar results were obtained when qPCR was performed as described for FAC-sorted cells^43^.

### *In vivo* physiology

After blood glucose levels had risen above ~20 mM (~2 days after tamoxifen injection), animals were immediately implanted subcutaneously with either a (1) placebo pellet, (2) 60-day slow-release glibenclamide pellet (17 mg kg^−1^ per day) (Innovative Research of America), or (3) two to three 30-day (~0.1 U per 24 h) insulin pellets (body weight <30 g, 2 pellets; >30 g, 3 pellets) (LinShin Canada Inc.) under 2% isoflurane anaesthesia. Placebo pellets contained vehicle but no drug. In separate experiments, Kir6.2-V59M expression was induced, mice left diabetic for 4 weeks (blood glucose >20 mM), then treated with glibenclamide (2-3 pellets; 34–95 mg kg^−1^ per day), and studied 4 weeks later.

Free-fed plasma glucose levels were measured daily (at 1400, h; FreeStyle Lite Blood Glucose Monitoring System, Abbott). Insulin and glucagon were measured in plasma or whole islets by radioimmunoassay (Millipore and Euro Diagnostica, respectively).

### Electrophysiology

Islets were isolated and dispersed into single cells, as described^12^. Single cells were cultured in 11 mM glucose and patched within 2 days of isolation. Na^+^ currents were recorded from cells within freshly isolated intact islets using the standard whole-cell configuration as described^44^ and following infusion with biocytin, cell identity confirmed by immunocytochemistry^45^. Macroscopic K_ATP_ currents were recorded from cell-attached membrane patches on isolated cells at −60 mV, filtered at 5 kHz and digitized at 20 kHz^12^. Cell identity was subsequently confirmed by immunocytochemistry.

### Electron microscopy

Isolated islets were fixed in 4% paraformaldehyde and 0.5% glutaraldehyde in phosphate buffer for 1 h, post-fixed in 1% osmium tetroxide, block stained in 2% uranyl actetate, dehydrated in graded ethanol and embedded in Spurr’s resin (Agar Scientific, Stansted, UK). Ultrathin sections (70 nm) were cut onto Ni^2+^ grids, contrasted with 2% uranyl acetate and lead citrate, and examined in a Jeol 1010 microscope (Welwyn Garden City, UK) with an accelerating voltage of 80 kV.

β-Cell apoptosis was assessed from electron micrographs as the number of β-cells with apoptotic nuclei (that is, displaying typical chromatin condensation): β-cells (135–150 from *n*=3–9 mice; *n*=3 islets per mouse) were identified by the presence of characteristic insulin granules.

### Immunostaining and morphometric analysis

Immunostaining was performed as described on fixed wax-embedded pancreatic sections^12^ and dispersed islet cells were fixed within 2 h of isolation. For antibodies see Supplementary Table 2. Insulin and glucagon area was analysed using the manual histology tool in Zeiss LSM-510 software. Five pairs of 5-μm-thick serial sections, 100 μm apart were analysed, for *n*=3–6 animals of each genotype (~160 islets/mouse).

### Flow cytometry

Pancreatic islet cell suspensions from β-RFP and βV59M-RFP mice were separated by FAC sorting using a MoFlo Beckman Coulter Cytomation sorter (Coulter Corp., Hialeah, FL). Islet populations were sorted immediately following isolation into RNAse-free collection buffer for qPCR analysis as described previously^45^. For Taqman probes, see Supplementary Table 3.

### Statistics

Data are mean values±s.e.m. of the indicated number of experiments. Significance was tested using One-way analysis of variance, Mann–Whitney Test and Bonferroni *post-hoc* test applied as indicated.

## Author contributions

M.F.B. and F.M.A. designed the study and wrote the manuscript. M.F.B., M.I., K.S., Q.Z., P.P., A.E.A., I.I.S., K.K.M., W.D., R.R. and A.C. performed experiments and analysed the data. M.F.B., F.M.A., F.M.G. and F.R. generated the β-RFP mice. P.R., F.M.G., F.R. and A.C. revised and edited the manuscript. Funding was provided by F.M.A., P.R., F.M.G. and F.R.

## Additional information

**How to cite this article:** Brereton, M. F. *et al*. Reversible changes in pancreatic islet structure and function produced by elevated blood glucose. *Nat. Commun.* 5:4639 doi: 10.1038/ncomms5639 (2014).

## Supplementary Material

Supplementary Information Supplementary Figures 1-6 and Supplementary Tables 1-3
