# Supplementary material for: Reversible changes in pancreatic islet structure and function produced by elevated blood glucose
Source: Nat Commun. 2014 Aug 22;5:4639. doi: 10.1038/ncomms5639 (PMC4143961; doi:10.1038/ncomms5639)
Supplement: Supplementary Information — Supplementary Figures 1-6 and Supplementary Tables 1-3 [file ncomms5639-s1.pdf]

Supplementary figure-1 Ashcroft

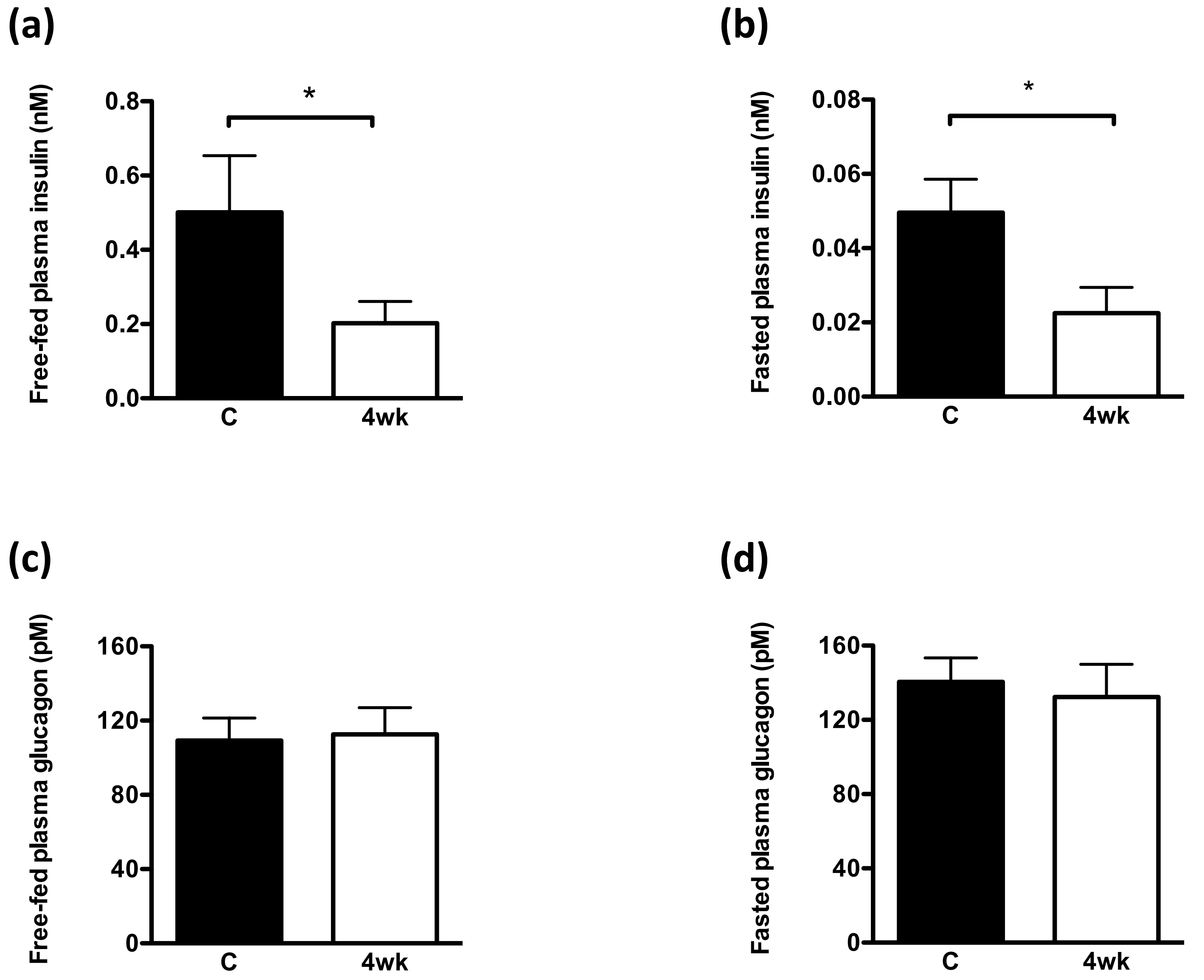

Supplementary figure 1: Plasma insulin and glucagon levels following gene induction

a,b. Mean±SEM free-fed (***a***) and fasted (***b***) plasma insulin levels for control mice (C, n=8-10) and 4-week-diabetic βV59M mice (4wk, n=6-9). ***c,d.*** Mean±SEM free-fed (***C***) and fasted (***D***) plasma glucagon levels for control mice (C, n=8-10) and 4-week-diabetic βV59M mice (4wk, n=6-9). **P*<0.05; Mann-Whitney test.

(a) Supplementary figure-2 Ashcroft

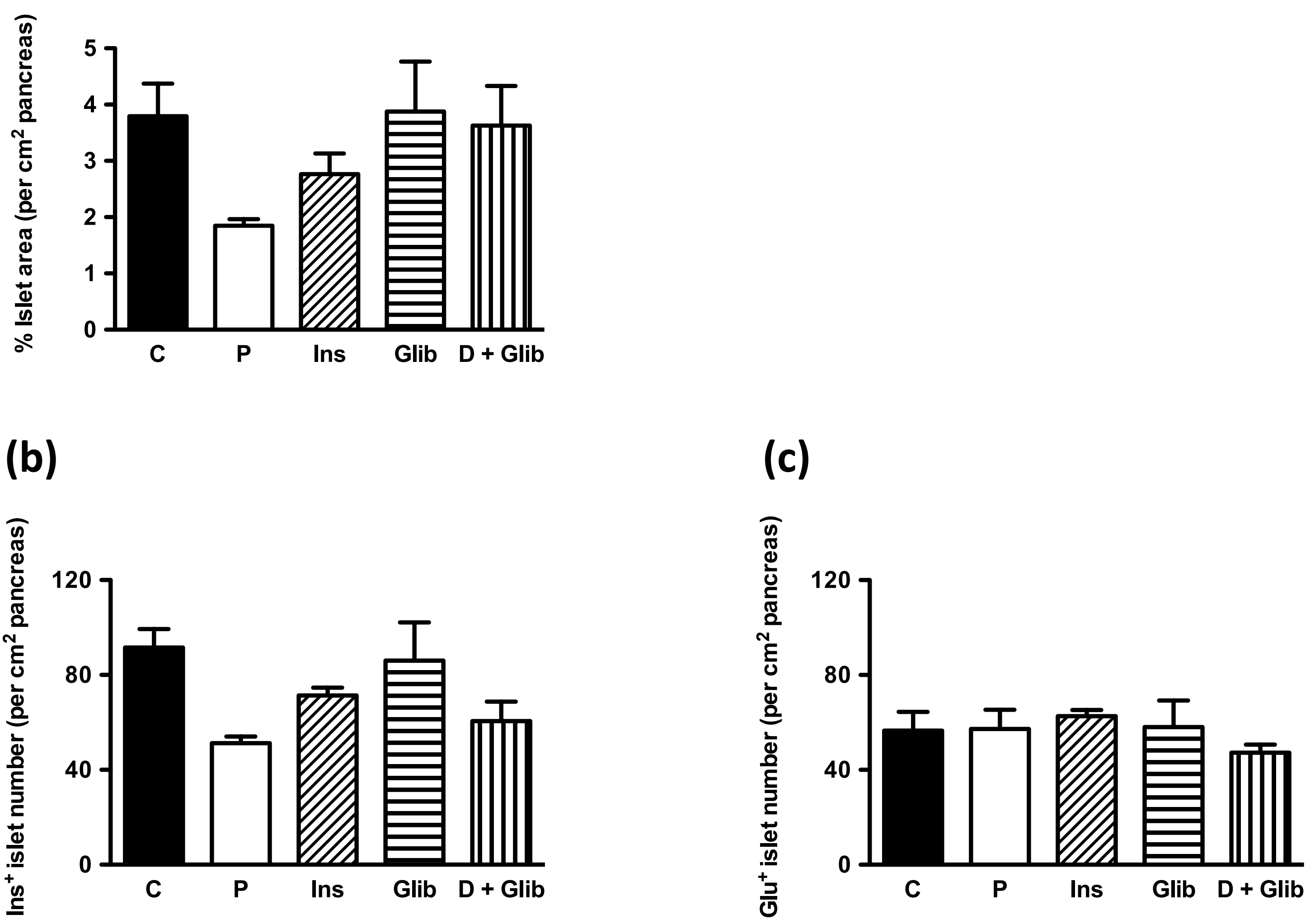

Supplementary figure 2: Chronic hyperglycaemia does not alter islet density

Islet area per cm^2 of pancreas (***a***) and number of insulin-positive (***b***) and glucagon-positive (***c***) islets (mean±SEM of 3-6 mice per genotype; P>0.05 compared to placebo; One-way ANOVA). After gene induction and elevation of blood glucose (< 20 mM) βV59M mice were implanted for 4 weeks with either placebo (P), insulin (Ins) or glibenclamide (Glib) pellets, or were implanted with glibenclamide pellets following 4 weeks of hyperglycaemia (P+Glib).

Supplementary figure-3 Ashcroft

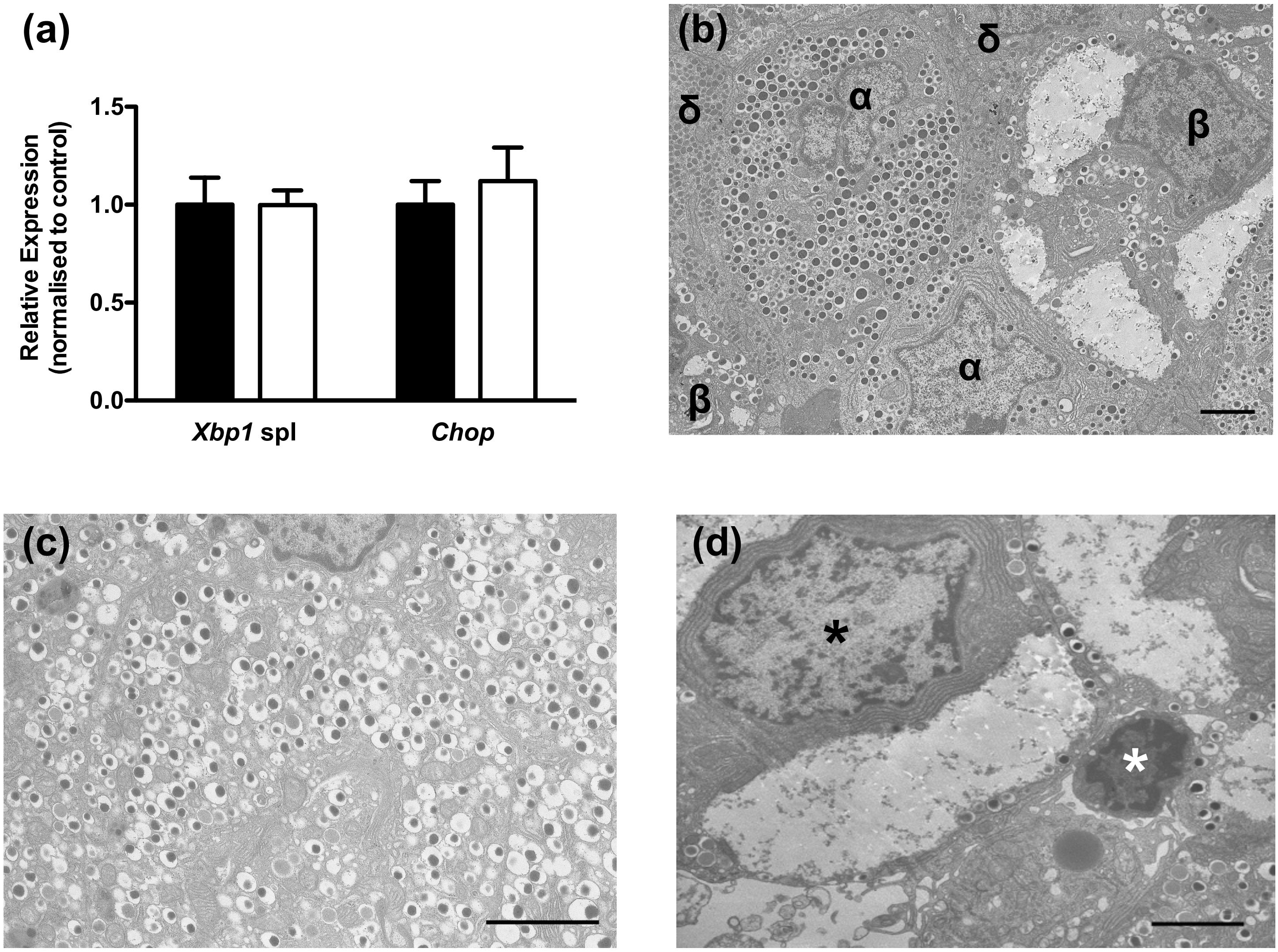

Supplementary figure 3: Effects of immediate and long-term gene induction on β-cell ultrastructure

a. *Xbp1* spliced and *Chop* mRNA levels in islets isolated from control (black bars) and 4-week-diabetic βV59M mice (white bars) (*P*>0.05; Mann-Whitney test; n=5-6 mice per genotype). Representative electron micrographs of pancreatic sections from βV59M mice exposed to hyperglycaemia for 4 weeks (***b***) and following gene induction and 24 h of established diabetes (***c;*** >20 mM glucose)**.** Alpha (α) beta (β) and delta (δ) cells are indicated. ***d.*** Electron micrograph of pancreatic section from a 4-week-diabetic βV59M islet with an example of an apoptotic nucleus with condensed chromatin (white *) compared to a non-apoptotic nucleus (black *). Scale bars 2μm.

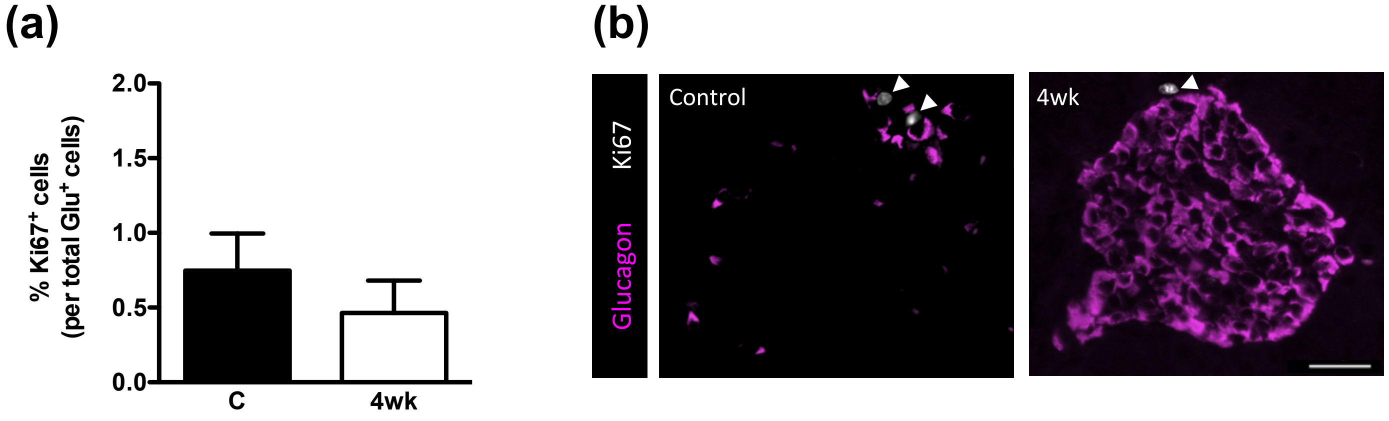

Supplementary figure 4: Chronic hyperglycaemia does not affect α-cell proliferation

a. Percentage of cells positive for both glucagon and Ki67, expressed as a percentage of the total number of glu$^+$ cells (*P*>0.05; Mann-Whitney test; n=3000-5000 glu$^+$ cells; n=130-190 islets; n=3-4 mice per genotype). ***b.*** Representative example of glu$^+$ cells (pink) and Ki67$^+$ nuclei (white arrow head) in control and 4-week-diabetic βV59M islets. Scale bar, 50µm.

Supplementary figure-5 Ashcroft

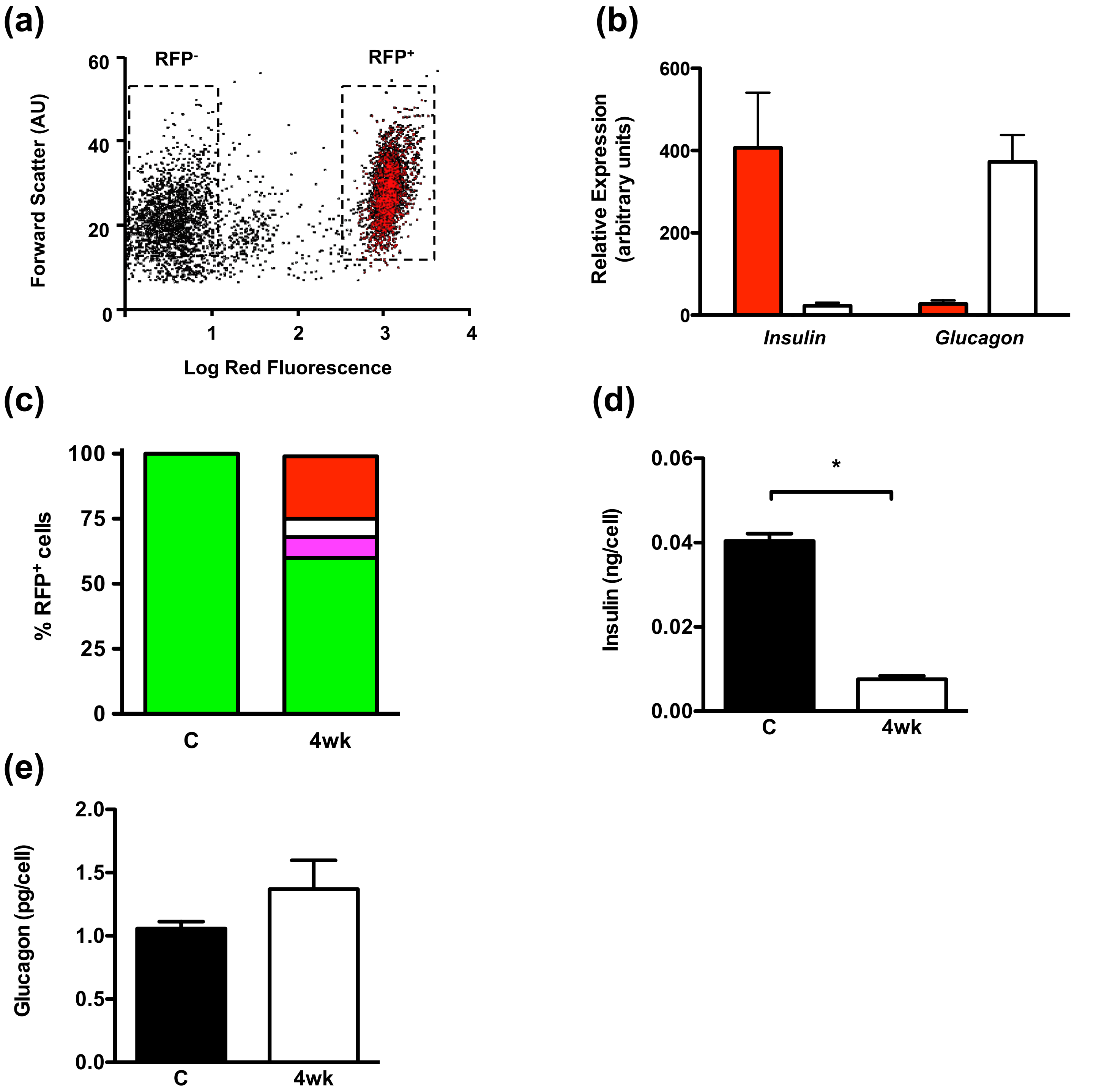

Supplementary figure 5: RFP fluorescence enables FAC sorting of pancreatic β-cells

Dispersed pancreatic islet cells from β-RFP and 4-week-diabetic βV59M-RFP mice were analysed by FAC sorting. (***a***) Representative example of a β-RFP FAC sort. Red fluorescence was measured by excitation at 560 nm and emission at 585/29 nm and plotted against forward scatter in arbitrary units (AU). Boxed areas indicate cells taken for analysis. ***b***. RFP^+ (black bars) and RFP^- (white bars) cells separated from β-RFP mice express insulin and glucagon respectively. ***c***. Quantification of the percentage of RFP^+ mouse islets that express insulin (green), glucagon (pink), insulin and glucagon (white), or do not contain detectable levels of either insulin or glucagon (red) in β-RFP (C) and 4-week-diabetic βV59M-RFP mice (4wk). (n=300-400 cells; n=3-4 mice per genotype). ***d,e.*** Insulin content of RFP^+ cells (***d***) and glucagon protein content of RFP^- cells (***e***) from β-RFP and 4-week-diabetic βV59M-RFP mice (mean ± SEM, P>0.05; Mann-Whitney test; n=4-5 mice per genotype).

Supplementary figure-6 Ashcroft

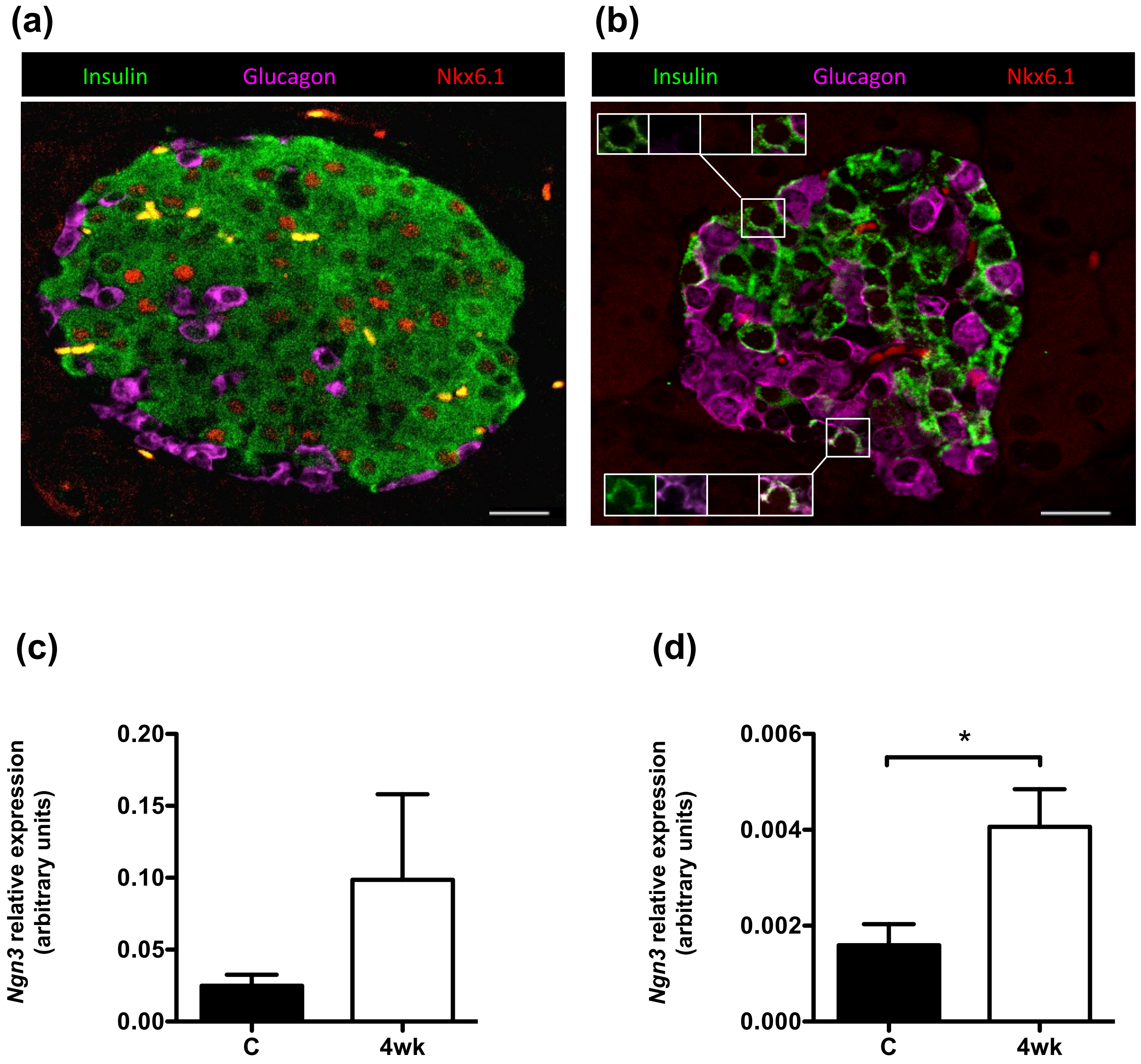

Supplementary figure 6: Transcription factor profile of diabetic βV59M islets.

a,b. Representative example of immunofluorescence staining for Nkx6.1 (red), insulin (green), and glucagon (pink) in a control islet. Scale bar 50µm. ***c,d.*** *Ngn3* mRNA levels in isolated islets (***c***) and FAC-sorted RFP^+ cells (***d***) from control mice (black bars) and 4-week-diabetic βV59M mice (white bars) (Mean ± SEM, * $P<0.05$; Mann-Whitney test; n=5-6 mice per genotype).

SYBR Green Primers	
**Gene**	**Sequence (5’ to 3’)**
Actb	F: GCAGCTCCTTCGTTGCCGGT<br>R: TACAGCCCGGGGAGCATCGT
Arx	F: TCCGGATACCCCACTTAGCTT<br>R: GACGCCCCTTTCCTTTAAGTG
CHOP	F: AGCCTGGTATGAGGATCTGCAG<br>R: GGTCAAGAGTAGTGAAGGTTTTTGATTC
Gfp	F: CGGCGACGTAAACGGCCACA<br>R: CAGCTTGCCGGTGGTGCAGA
Glucagon	F: ACCTGGACTCCCGCCGTGC<br>R: TCGCCTTCCTCGGCCTTTCACCAGCC
Glut2	F: TCTTCACGG CTGTCTCTGTG<br>R: AATCATCCCGGTTAGGAACA
Hprt1	F: CCGGCAGCGTTTCTGAGCCA<br>R: GCTCGCGGCAAAAAGCGGTC
Hspa8	F: CAGCGCAGCTGGGCCTACAC<br>R: TAGCTTGGCGTGGTGCGGTT
Insulin	F: GCATCCSGTAACCCCCAGCCC<br>R: CAGGGCCAGCAGGGGTAGGA
MafA	F: CAGCAGCGGCACATTCTG<br>R: GCCCGCCAACTTCTCGTAT
MafB	F: AGGACCTGTACTGGATGGC<br>R: CACTACGGAAGCCGTCGAAG
Ngn3	F: TCTCAAGCATCTCGCCTCTTC<br>R: ACAGCAAGGGTACCGATGAGA
Nkx6.1	F: AGAGAGCAGGCTTGGCCTATTC<br>R: GTCGTCAGAGTTCGGGTCCAG
Pax6	F: AAACAAACGCCCTAGCTCTCC<br>R: CCGCCCTTGGTTAAAGTCCTC
Pdx1	F: CAGTGGGCAGGAGGTGCTTA<br>R: GCCCGGGTGTAGGCAGTAC
Xbp1 spliced	F: CTGAGTCCGAATCAGGTGCAG<br>R: GTCCATGGGAAGATGTTCTGG

Supplementary Table 1: SYBR green primer sequences for the indicated genes.

**Antibody**	**Concentration**	**Source**
Guinea pig anti-insulin	1:500	Made in house
Mouse anti-glucagon	1:500	Sigma (2645)
Mouse anti-Pdx1	1:50	DHDB (F6A11)
Rabbit anti-Ki67	1:50	Thermo Scientific (RM 9106S1)
Rabbit anti-dsRed	1:100	Clontech (632496)
Anti-guinea pig HRP	1:50	Dako (P0141)
Anti-guinea pig Alexa 633	1:200	Molecular Probes (A21105)
Anti-guinea pig Texas red	1:50	Vector Laboratories (TI-7000)
Anti-mouse Alexa 633	1:500	Molecular Probes (A-21046)
Anti-mouse Cy3	1:500	Jackson Laboratories
Anti-mouse Alexa 488	1:200	Jackson Laboratories
Anti-mouse HRP	1:50	Vector Laboratories (PI-2000)
Anti-rabbit Alexa 568	1:200	Molecular Probes (A11037)
Streptavidin Alexa 488	1:500	Molecular Probes (S11223)
Rabbit-anti MafA	1:50	Bethyl Laboratories
Rabbit anti-MafB	1:25	Bethyl Laboratories
Rabbit anti-Glut2	1:15	Santa Cruz (sc-9117)
Rabbit anti-glucagon	1:1000	Abcam (ab92517)
Mouse-anti-Nkx6.1	1:50	DHDB (F55A12-c)

Supplementary Table 2: Antibodies.

**Taqman Primers**	
**Gene**	**Sequence (5' to 3')**
Arx	Mm00545903_m1 (Applied Biosystems)
Beta actin	Mm00607939_s1 (Applied Biosystems)
Glucagon	Mm01269055_m1 (Applied Biosystems)
Glut2	Mm00446224_m1 (applied Biosystems)
Insulin	Primer 1: AACCCACCCAGGCTTTTGTCA<br>Primer 2: CTTCCTCCCAGCTCCAGTTGTTC<br>Probe: GGCTCTCTACCTGGTGTGTGGGGAGCGT
MafA	Mm00845209_s1(Applied Biosystems)
MafB	Mm00627481_s1 (Applied Biosystems)
Ngn3	Mm00437606_s1 (Applied Biosystems)
Nkx6.1	Mm00454962_m1 (Applied Biosystems)
Pdx1	Mm00435565_m1 (Applied Biosystems)

Supplementary Table 3: Taqman primer sequences for the indicated genes.
